# 3D MRI of explanted sheep hearts with submillimeter isotropic spatial resolution: comparison between diffusion tensor and structure tensor imaging

**DOI:** 10.1007/s10334-021-00913-4

**Published:** 2021-02-27

**Authors:** Julie Magat, Valéry Ozenne, Nicolas Cedilnik, Jérôme Naulin, Kylian Haliot, Maxime Sermesant, Stephen H. Gilbert, Mark Trew, Michel Haissaguerre, Bruno Quesson, Olivier Bernus

**Affiliations:** 1 IHU Liryc, Electrophysiology and Heart Modeling Institute, Hopital Xavier Arnozan, 33600 Pessac, France; 2 Centre de Recherche Cardio-Thoracique de Bordeaux, U1045, University of Bordeaux, 33000 Bordeaux, France; 3 INSERM, Centre de Recherche Cardio-Thoracique de Bordeaux, U1045, 33000 Bordeaux, France; 4 Epione researc team INRIA, Sophia antipolis, France; 5 Mathematical Cell Physiology, Max Delbrück Centre for Molecular Medicine in the Helmholtz Association, 13125 Berlin-Buch, Germany; 6 Department of Physiology, Bioengineering Institute, University of Auckland, Auckland, New Zealand; 7 Electrophysiology and Ablation Unit, Bordeaux University Hospital (CHU), 33600 Pessac, France

**Keywords:** Cardiac microstructure, Fiber organization, Sheetlet organization, High-resolution MRI, Helix angle, Structure tensor

## Abstract

**Objective:**

The aim of the study is to compare structure tensor imaging (STI) with diffusion tensor imaging (DTI) of the sheep heart (approximately the same size as the human heart).

**Materials and methods:**

MRI acquisition on three sheep ex vivo hearts was performed at 9.4 T/30 cm with a seven-element RF coil. 3D FLASH with an isotropic resolution of 150 µm and 3D spin-echo DTI at 600 µm were performed. Tensor analysis, angles extraction and segments divisions were performed on both volumes.

**Results:**

A 3D FLASH allows for visualization of the detailed structure of the left and right ventricles. The helix angle determined using DTI and STI exhibited a smooth transmural change from the endocardium to the epicardium. Both the helix and transverse angles were similar between techniques. Sheetlet organization exhibited the same pattern in both acquisitions, but local angle differences were seen and identified in 17 segments representation.

**Discussion:**

This study demonstrated the feasibility of high-resolution MRI for studying the myocyte and myolaminar architecture of sheep hearts. We presented the results of STI on three whole sheep ex vivo hearts and demonstrated a good correspondence between DTI and STI.

**Supplementary Information:**

The online version contains supplementary material available at 10.1007/s10334-021-00913-4.

## Introduction

Myocardial structure plays an important role in the normal function of the heart, by facilitating efficient contraction and significantly influencing electrical propagation [1]. Various cardiac diseases are associated with major structural remodeling, which impacts cardiac function.

The myocardium consists of branching myolaminae, or sheetlets, which are 4–6 cells thick. The long axis of the myocytes within these laminae, also referred to as fiber orientation, shows a regular low-order organization in the ventricular wall often described as a helical transmural arrangement, which was described previously in detail [2–4]. The laminar architecture, i.e., the orientation of the myolaminae, is, however, more complex, with rapidly branching sheetlets separated by interstices containing collagen bundles [5]. Both myofibers and myolaminar architecture are present throughout the myocardium and exhibit orthotropy along three orthogonal directions along the fiber axis, perpendicular to the fibers in the sheetlet plane, and normal to the sheetlet plane.

Various techniques have been used to visualize the myocardial architecture, including histology [6], extended confocal imaging (ECI) [7] and magnetic resonance imaging (MRI). Histology provides high-resolution (HR) images of the cardiac structure, but it is a destructive technique with limited three-dimensional (3D) volumetric reconstruction and spatial coverage, a limitation that also applies to ECI.

Different MRI techniques can provide information on myocardial–myocyte orientation and myolaminar/sheetlet structure in the intact heart. Over the last 15 years, diffusion tensor imaging (DTI) has been applied to investigate cardiac fiber orientation in vivo [8–10] and ex vivo in 3D [11, 12]. Several species have been studied, including rodents [2, 11], dogs [13], sheep [14], pigs [9], and humans [15]. In 2003, Köhler et al. compared diffusion-weighted MRI and T2* images, and found that there was a good agreement in the visualization of microstructure in isolated rat hearts [16]. The technique was also validated by comparison with histology [17]. Following from this earlier work, Gilbert et al. proposed and validated 3D HR MRI acquisition at an isotropic resolution of 50 µm^3^ to visualize and quantify cardiac microstructure in ex vivo rat hearts [18]. They demonstrated that structure tensor imaging (STI) at an isotropic resolution of 200 µm^3^ provided accurate information on sheetlet orientation and cardiac orthotropy and was superior to DTI [19]. Our group recently reported preliminary results on STI and DTI of one human heart [20]. However, it was only applied on a single heart, and requires further validation on post processing and visualization in large mammalian species.

The aim of this study was to present a pipeline acquisition of DTI and STI, and compare the two modalities to assessing cardiac microstructure by analysis fiber orientation and sheetlet structure on three ex vivo sheep hearts at 9.4 T with a large bore access of 30 cm.

## Materials and methods

### Sample preparation

Hearts (hearts 1–3) were explanted from three female sheep (weight: ~ 50 kg) via sternal thoracotomy under general anesthesia. This protocol was approved by the Animal Research Ethics Committee in accordance with the European rules for animal experimentation. The three hearts (~ 12 × 8 × 6 cm^3^, heart weight = 150 ± 10 g) were perfusion-fixed in 10% formaldehyde (total fixation solution of 1 L) containing 2 mL of gadoterate meglumine (Gd Dotarem), a gadolinium-based contrast agent (Dotarem; Guerbet, Paris, France) for 12 h. This method provides enhanced contrast between the myolaminae and cleavage planes and a greater signal-to-noise ratio [18]. Then, hearts were placed inside a container filled with perfusion solution and were stored in a cold room (4 °C). For MRI acquisition, first samples were removed from the solution and stored in a plastic container.; second, using a 10-mL syringe, the heart cavities were carefully filled with Fomblin (Solvay Solexis, Brussels, Belgium), which is a perfluoropolyether with no 1H detectable signal when scanned using MRI. It offers similar magnetic susceptibility to tissue such that it attenuates susceptibility artifacts at the border of the cardiac chambers [18]. Finally, whole hearts immersed in Fomblin were sealed in a plastic container.

### MRI

All experiments were performed at 9.4 T (400 MHz resonance frequency for the water protons) with an open bore access of 30 cm (BioSpin MRI; Bruker, Ettlingen Germany), a 200-mm inner diameter gradient (300 mT/m), and a shim system. Hearts were placed in the center of the magnet in the base–apex axis along the gradient coil.

A dedicated RF volume coil for imaging large samples designed by Bruker was used for scanning. The RF coil was a cylindrical volume array (inner/outer diameters of 165/198 mm, respectively) including seven equally spaced overlapping loop elements (100 mm width, 175 mm length). In this design, no exactly opposing elements occur, but each element is opposed to a gap between two other elements, thus strongly reducing the degree of coupling between the critical elements without the need of reducing the loop size. Phase setting of each element was shifted of 45°–50° to avoid destructive interference. Then, scout images were obtained, 3D B1 maps were acquired within 20 min using the Bloch Siegert shift [21] method for all RF coil elements with a gradient echo sequence modified by including an off-resonance radiofrequency pulse with an offset of 6 kHz relative to water. Based upon preliminary work and our experimental experience [20], a 3D shim box was placed in the septum. Local B1+ shimming was performed by determining a set of transmit phases and amplitudes to maximize the homogeneity within the region of interest (ROI).

### 3D DTI

Diffusion tensor MRI was carried out using a 3D diffusion-weighted spin-echo sequence with TE = 22 ms, TR = 500 ms, FOV = 100 × 80 × 110 mm, matrix = 166 × 133 × 183 and an isotropic resolution of 600 μm. Each diffusion gradient had a duration of 4.5 ms, and gradients were separated by an 11-ms delay: three b0 maps were generated and six gradient directions were applied with a *b* value of 1000 s/mm^2^, as described previously [19], and an partial Fourier undersampling of 1.8 factor in phase direction was used, for a total acquisition time of 16 h 55 min. Raw diffusion-weighted images were processed using ParaVison 6.0 (Bruker) to compute the diffusion tensor.

**Fig. 1:**
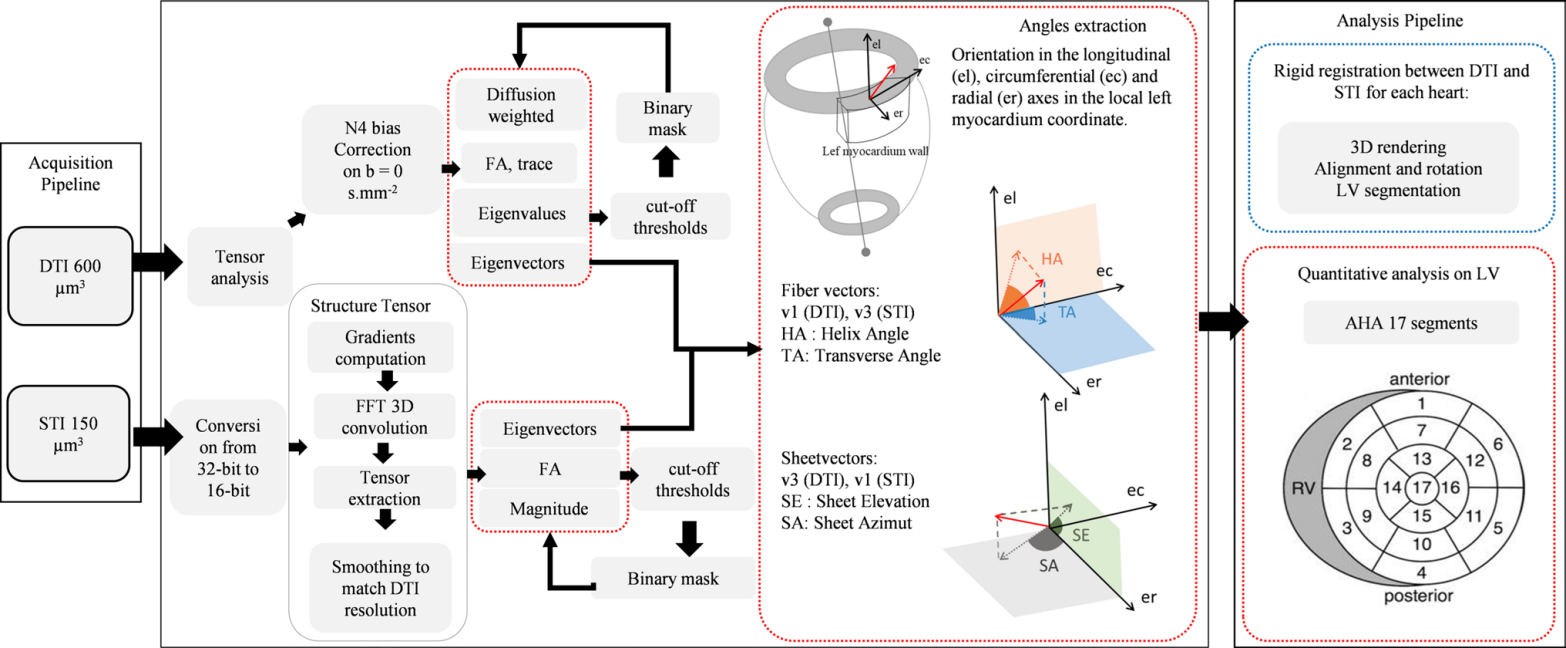
Pipeline of magnetic resonance imaging (MRI) data acquisition, post-processing, and analysis for each sample inspired from [20]. Diffusion tensor (DT) spin-echo and structure tensor (ST) gradient-echo data were processed through two different pipelines yielding the helix angle (HA), transverse angle (TA), sheetlet elevation (SE) angle, and sheetlet azimuth (SA) angle. The transformation to align the long axis of the LV along the *z* axis was applied on angles maps. The angle maps were then registered along the short axis using rigid registration, the left ventricle (LV) was segmented, and the 17-segment American Heart Association (AHA) model was applied to quantitatively analyze 17 segments of the mid-LV.

For segmentation of the cardiac ventricles, N4 bias correction [22] was applied on maps with *b* = 0 s/mm^2^ to avoid the segmentation of regions showing some B1 inhomogeneity and signal drop-off associated with RF coil sensitivity profile in the region of the cardiac apex and base (Fig. 1, processing pipeline). Low and high cutoff thresholds were applied to the FA, trace, and weighted images to define a binary mask. The first diffusion tensor eigenvector has been shown to correspond to myocyte orientation DTI [6–9]. The second and third eigenvectors have been associated with sheetlet in-plane and normal directions, respectively, but their accuracy in determining tissue laminar organization is poor [19]. Computer post processing to extract the tensor and vectors from DTI acquisitions lasted less than 1 min.

### 3D STI

A 3D FLASH sequence was applied to image the whole heart volume, which was averaged 20 times, with the following settings: TE = 9 ms; TR = 30 ms; matrix size = 731 × 665 × 532; FOV = 110 × 100 × 80 mm, voxel dimensions = 150 µm isotropic, flip angle = 30°, and an accelerator factor in phase direction of 1.91. The total acquisition time for each heart was 30 h 50 min.

T1-weighted images contrast was used to determine the orientation of the sheetlet and myofibers. The following steps were applied to the FLASH images:

- Images were reformatted in short axis view and converted into a stack of 16-bit images (Fig. 1, processing pipeline).
- The derivatives of the image intensity (the intensity gradient) along each direction were computed: first a 5 × 5 × 5 point derivative template was defined [23]. The derivative template was applied on the full 3D images using 1D FFT convolution as described previously [18, 19]. The 5 × 5 × 5 kernel applied to 150 µm isotropic images involved a region of 750 × 750 × 750 µm. This resolution represents the size of local environment that contributes to the structure tensor at a given point.”
- The structure tensor (the outer product of the intensity gradient vectors) was computed for each voxel and smoothed at a progressive resolution from 150 to 600 µm isotropic (731 × 665 × 532 tensors to 183 × 166 × 133 tensors). Each structure tensor component at resolution doubling was determined using level 4 binomial low-pass filters to smooth from one level of resolution to the next.
- The principal directions of the structure tensor at each discrete point were extracted using eigenanalysis at isotropic resolution 600 µm. The 600 µm smoothed structure tensor data set was used to best-match the expected diffusion tensor resolution. (See steps in Fig. 1).
- We used a previously described tissue coordinate reference frame [18, 19] as follows: the first eigenvector (largest magnitude eigenvalue) corresponds to the sheetlet/lamina normal direction, the second to the sheetlet/lamina in-plane direction, and the third (smallest magnitude eigenvalue) to the myocyte orientation.
- Additionally, binary masks were created based on FA, trace, and image intensity to segment the background using low and high cutoff thresholds.

Post data treatment after acquisition to reconstruct images, and extract tensors and eigenvector lasted 1 h. The processing pipeline was implemented [19, 20] using Matlab (The MathWorks Inc., Natick, MA) and VTK libraries.

### Comparison between DTI and STI

Our previous study [19] in rat hearts showed that DTI provides robust information on fiber orientation, whereas STI is more accurate in terms of sheetlet orientation. A mean of 897,181 ± 32,096 voxels was extracted for the three hearts using DTI, and 798,645 ± 33,051 voxels from FLASH images were post-processed to obtain the same resolution as DTI (isotropic resolution of 600 µm with 133 × 167 × 183 tensors).

As described previously [24], all structural information was obtained using a specific cardiac reference system (see Fig. 1 angles extraction), with an apex–base left ventricle (LV) axis running through the center of the left ventricular cavity. For each voxel in the segmented datasets, the orientations of myocytes (fibers) and sheetlets were computed in this coordinate system.

For myocyte orientation, we compared the first DTI eigenvector with the third STI eigenvector and computed the fiber helix angle (HA), which is the angle between the short axis plane and the projection of the fiber vector onto the wall tangent plane. The fiber transverse angle (TA) is the angle between the wall tangent plane (also known as the longitudinal orientation) and the projection of the fiber vector onto the transverse (also known as the short axis plane) (see Fig. 1 in angles extraction part).

For sheetlet orientation, the first STI eigenvector was compared to the third DTI eigenvector, which is assumed to be positioned normal to the sheet. The sheetlet elevation (SE) angle is the angle between the short axis plane and the projection of the vector onto the radial orientation. The sheetlet azimuth (SA) angle is the angle between the local radial orientation and the projection of the vector onto the short axis plane [11].

Each segment for each sample contains a mean of 16,032 ± 2270 voxels.

Mean transmural evolution was plotted (bin of 0.005, i.e., 200 points displayed) for each angle for 17 segments between the endocardium (normalized distance *x* = 0) and epicardium (normalized distance *x* = 1). We performed a local unwrap for pixels up to 90°.

### Registration and segmentation (Fig. 1 analysis pipeline)

Short axis registration of the parametric volume maps was performed. Rigid registration using 3D Slicer (http://www.slicer.org) [25] between DTI and STI for the same heart was performed to obtain the same configuration for both acquisitions. Data from STI were then aligned across the three samples to obtain a similar alignment among hearts. The LV was manually segmented, and quantitative transmural maps were subdivided into regions defined by the 17-segment American Heart Association (AHA) model [26]. We used a polar coordinate system described previously [27], in which segments were defined as ranges of angles.

### Statistical analysis and Image visualization and quantification

Scatter and Bland–Altman plots in whole three hearts using GraphPad Prism (GraphPad Software, San Diego, CA) were performed. Mean of HA angles for DTI and STI in endocardium, mid-ventricular and epicardium were calculated to analyze the reproducibility of measured between samples and similarities between methods. Statistical analysis are performed for comparison between groups using nonparametric test (Kruskal–Wallis test) with *p* < 0.05 are considered as statistically significant. One asterisk (*) identifies *p* values between 0.01 and 0.05, two asterisks (**) identify adjusted *p* values between 0.01 and 0.001.

Descriptive statistics from linear regression (*R*^2^ linearity and slope) and curve fitting were calculated for HA and TA transmural profile.

Bullseye representations were created using homemade program on Matlab after 17 AHA segmentation. Mean in each segment for the three hearts was visualized for DTI and STI results for HA, TA, SE and SA angles.

Image reconstruction was performed using ParaVision 6.0 (Bruker) on a workstation with 512 GB of accessible memory to process the large data matrices. Visualization of 3D volume renderings was performed using Volview and Paraview software (Kitware, Clifton Park, NY). Short axis registration of the parametric volume maps was performed using 3D Slicer software and (http://www.slicer.org) [25] and parametric maps were displayed using Advanced Normalization Tools (ANTS) libraries [28].

## Results

**Fig. 2:**
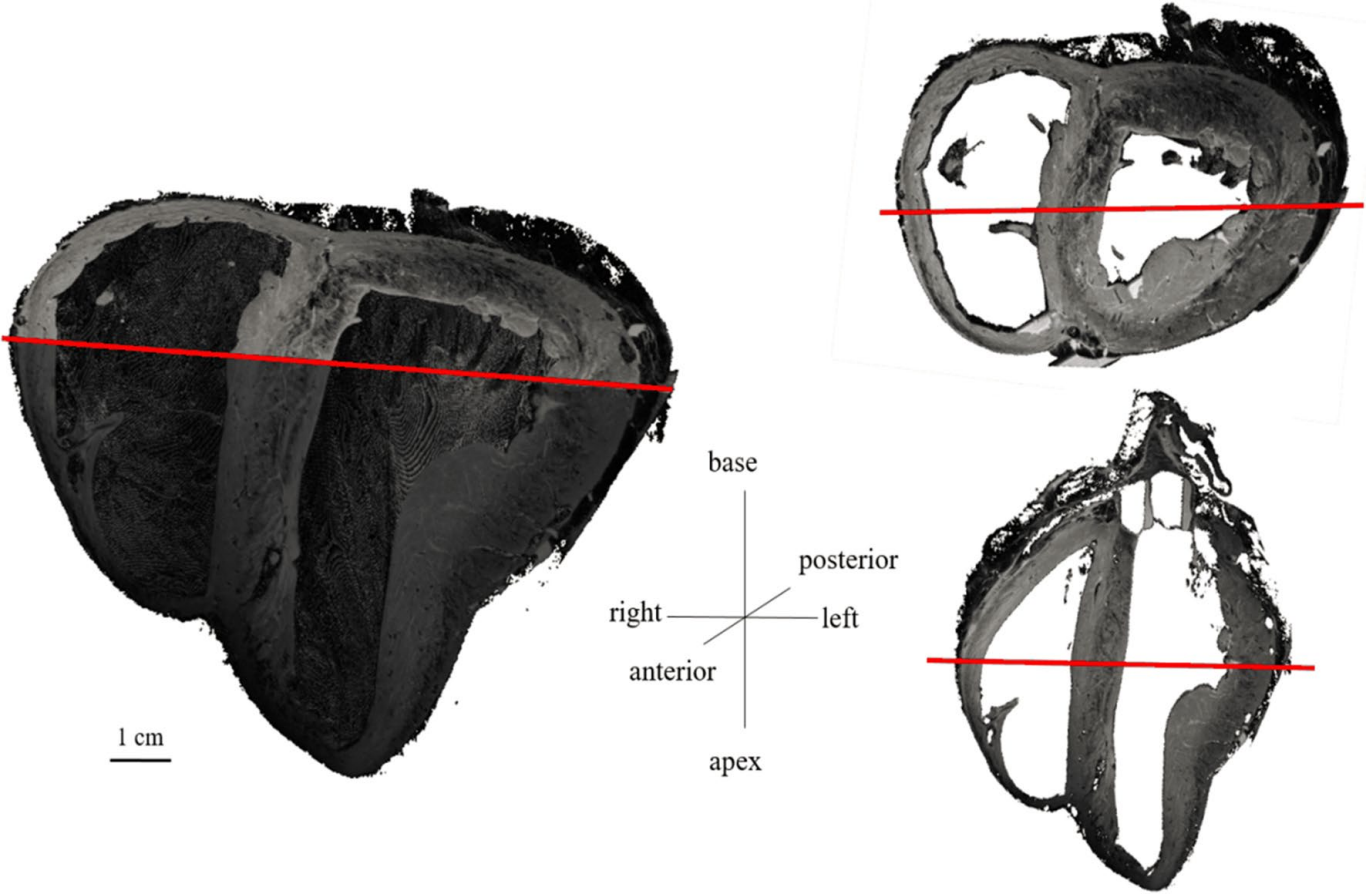
Three-dimensional (3D) high-resolution FLASH images (with an isotropic resolution of 150 µm) of one heart. On the left, volume rendering cropped by removing the heart base and anterior cavities. On the right, short axis and long axis views

**Fig. 3:**
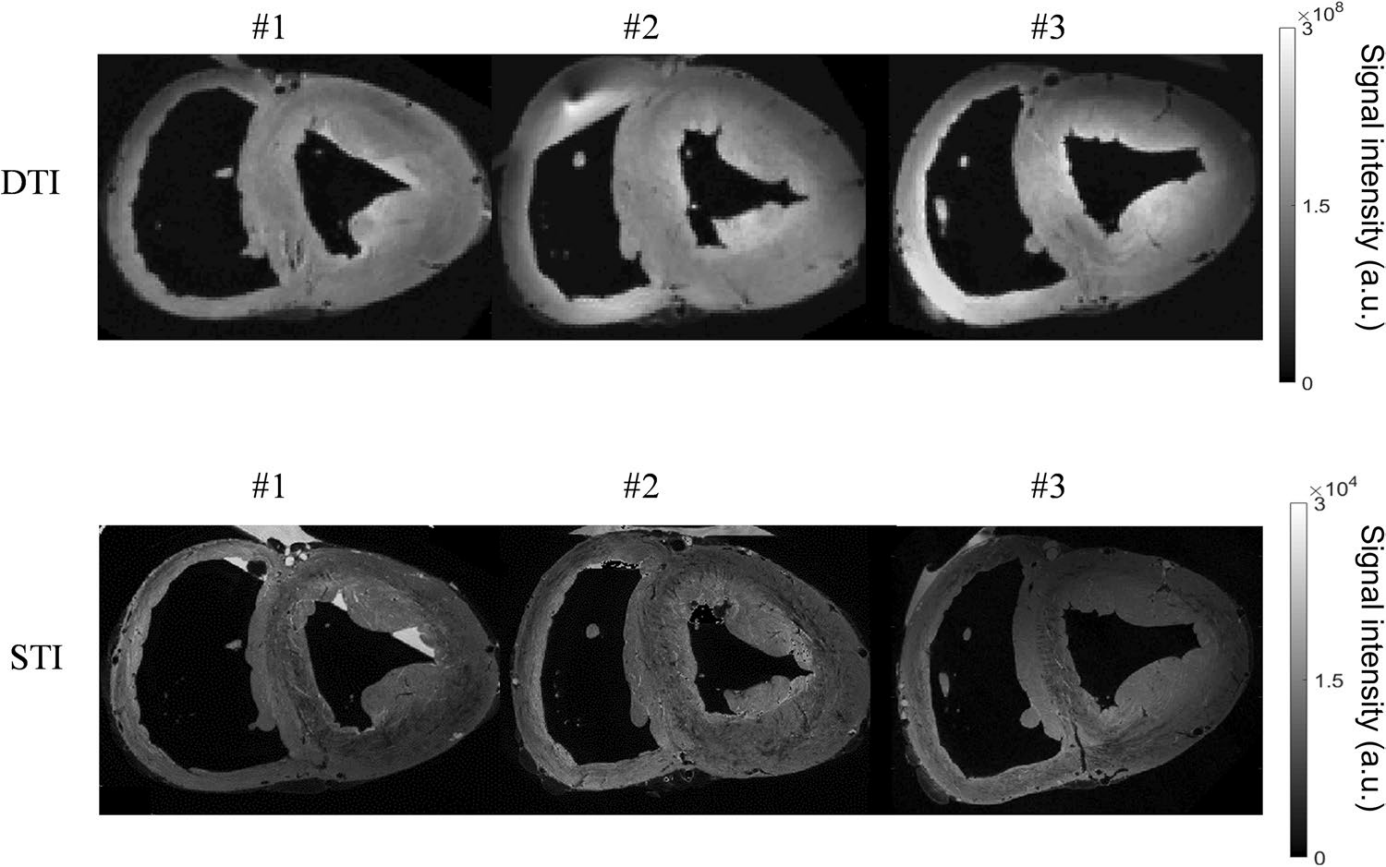
Raw images obtained from three sheep hearts (#1 to #3). First line, *b* = 0 image intensity (a.u.) from DTI acquisition at an isotropic resolution of 600 µm. Second line, FLASH images intensity (a.u.) at an isotropic resolution of 150 µm short axis views

Figure 2 shows a 3D volume rendering and short and long axis slices of one of the whole hearts using contrast-enhanced FLASH acquisitions at an isotropic resolution of 150 μm^3^. In these images, the interstices between sheetlets, as well as vessels, appear bright due to the presence of residual Gd Dotarem. The fine details of the laminar architecture (see animated gif in https://github.com/valeryozenne/Cardiac-Structure-Database/tree/master/Article) can be seen in these images. Raw images extracted from 3D volumes after MRI acquisitions are displayed in Fig. 3 for the three hearts (hearts #1 to #3) in short axis view. The first line shows diffusion-weighted images from DTI acquisition recovered at an isotropic resolution of 600 µm. The second line presents high-resolution images at 150 µm obtained after STI acquisitions. In term of samples fixation right and left ventricles are fixed in relaxed phase, no damages or scars are observed. For STI images we can identify same structures inside the left ventricular cardiac tissue with differences of fibers orientation from the endocardium to epicardium. A bright signal in the top of the samples appears in some acquisitions, it corresponds to a residue of formaldehyde and was removed by segmentation in post-processing data pipeline. Some residues from formalin trapped in the coronaries or vessels rise to the surface and can be observed as super intense signal on images. In heart #2, a bubble is trapped between right cavities, formalin and fomblin.

**Fig. 4:**
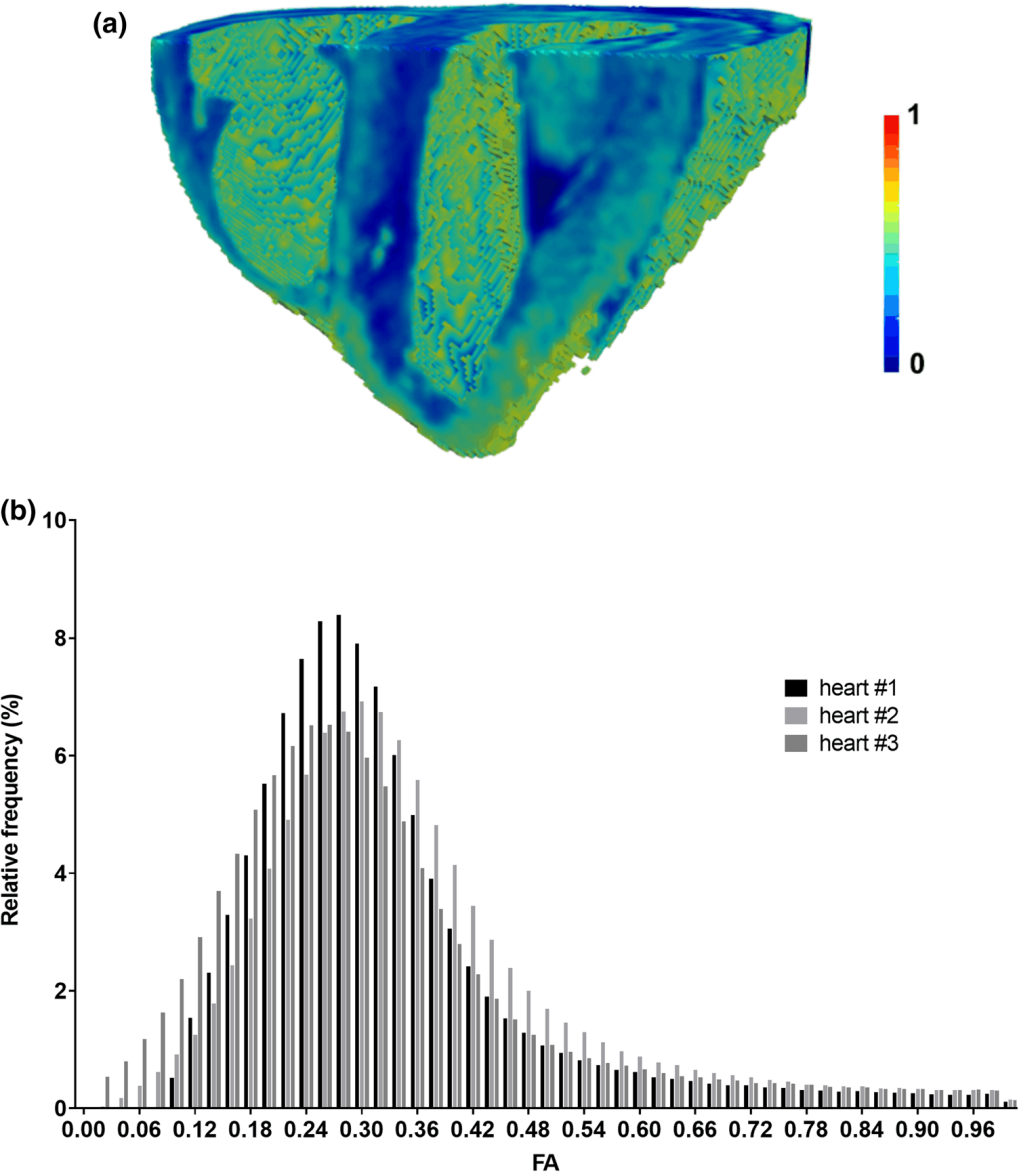
**a** Fractional anisotropy **(**FA) values extracted from the 3D DT analysis for each heart. FA volume rendering cropped for heart #1. **b** Cumulative histogram (% FA) corresponding to the whole heart for each sample

The tissue FA for the first heart is extracted from the 3D DTI acquisitions. Yellow and red areas are more anisotropic than blue areas (Fig. 4a). Figure 4b shows cumulative histograms of FA (binning of 0.02 between 0 and 1 ) extracted from DTI for the three hearts by segmenting out the cardiac bases and cavities. The graph reveals mean FA values of 0.33 ± 0.16, 0.3 ± 0.17, and 0.32 ± 0.18 for samples 1, 2, and 3, respectively, in the ventricular myocardium.

**Fig. 5:**
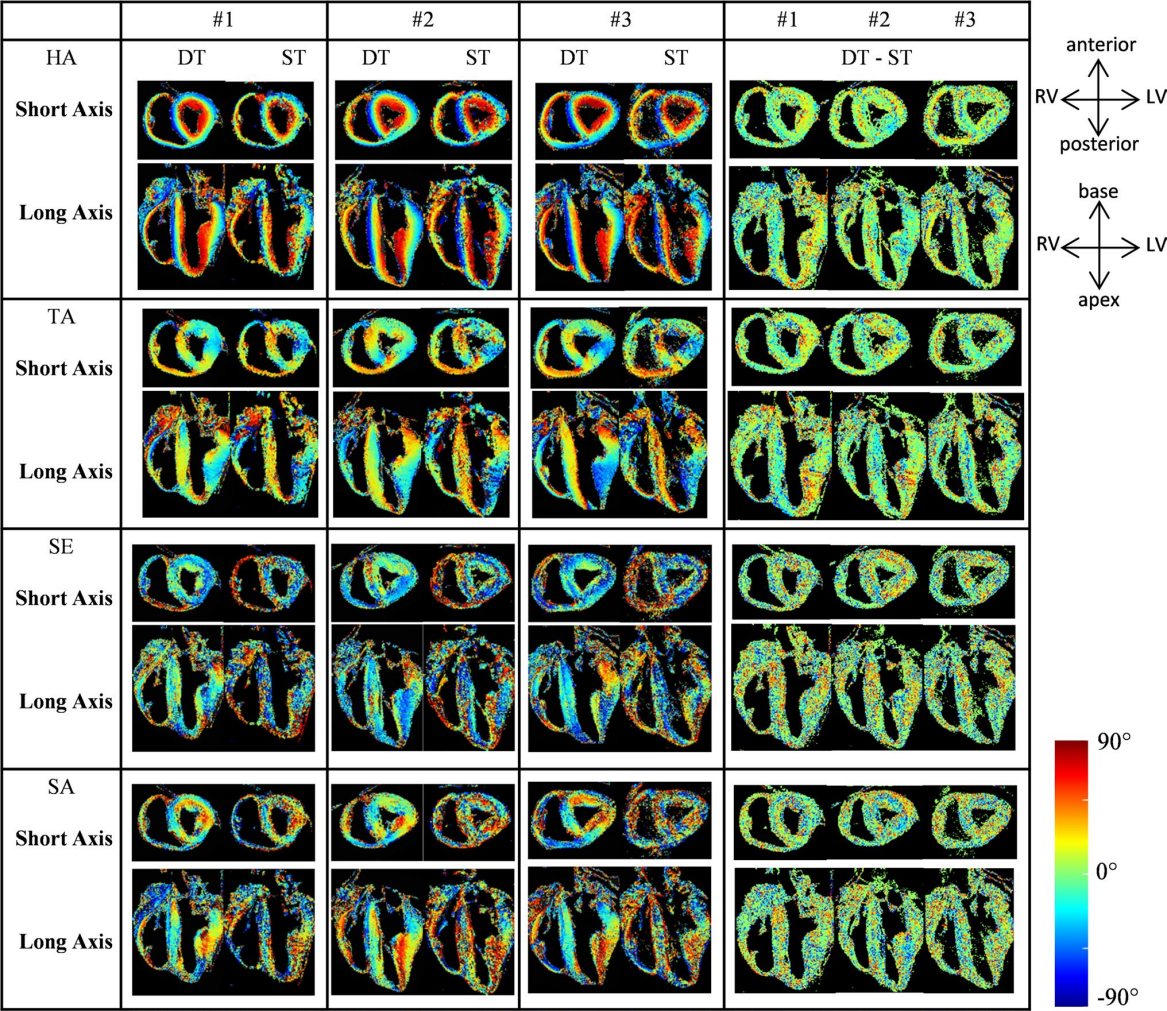
HA, TA, SE and SA angles between − 90° and 90° from DT imaging (DTI) and ST imaging (STI) after post-processing in short axis and long axis views for hearts #1, #2 and #3—with an isotropic resolution of 600 µm. Cut planes in the images are similar to Fig. 2

HA, TA, SE and SA maps are represented in Fig. 5 for representative short and long axis slices for each heart (hearts #1 to #3).

A qualitative interpretation of angles maps is presented in the following part: maps are consistent across the three hearts. In general, a larger noise level is observed in the STI maps. We identify the well-known transmural profile in the LV, where the HA varied smoothly from a negative value (blue–green) at the epicardium to a positive value (red) at the endocardium. There is a smooth transition between the RV and LV in the posterior position, and an abrupt change of orientation in the same area in the anterior view.

TA maps are mainly close to zero in short axis view. The angle distribution appeared to be more heterogeneous in the septum. TA maps derived from DTI and STI appear to be similar; short axis images of the left free wall for all hearts for both acquisitions showed negative values in the same areas.

There are similarities between STI and DTI for images of the LV, with SE angles < 0° in the LV and septum. In long axis views, the SE angle is positive at the apex and negative at the base for the three hearts. On SA angle maps, STI and DTI data are similar with abrupt angle changes in the LV in short axis view but heterogeneous angle distribution in the long axis view in the septum. In the RV, some differences can be seen between DTI and STI, with negative values derived from DTI and positive values derived from STI in short axis view, especially for SE angles in all samples. Abrupt changes in short axis orientation were seen in the ventricles and also at the LV–septum–RV insertions.

Difference angles maps between DT and ST images are represented in the last column of Fig. 5, global values are close to 0° with some local discrepancies especially in the septum for HA and TA maps. Differences maps for SE and SA display more negative values in short and long axis views.

**Fig. 6:**
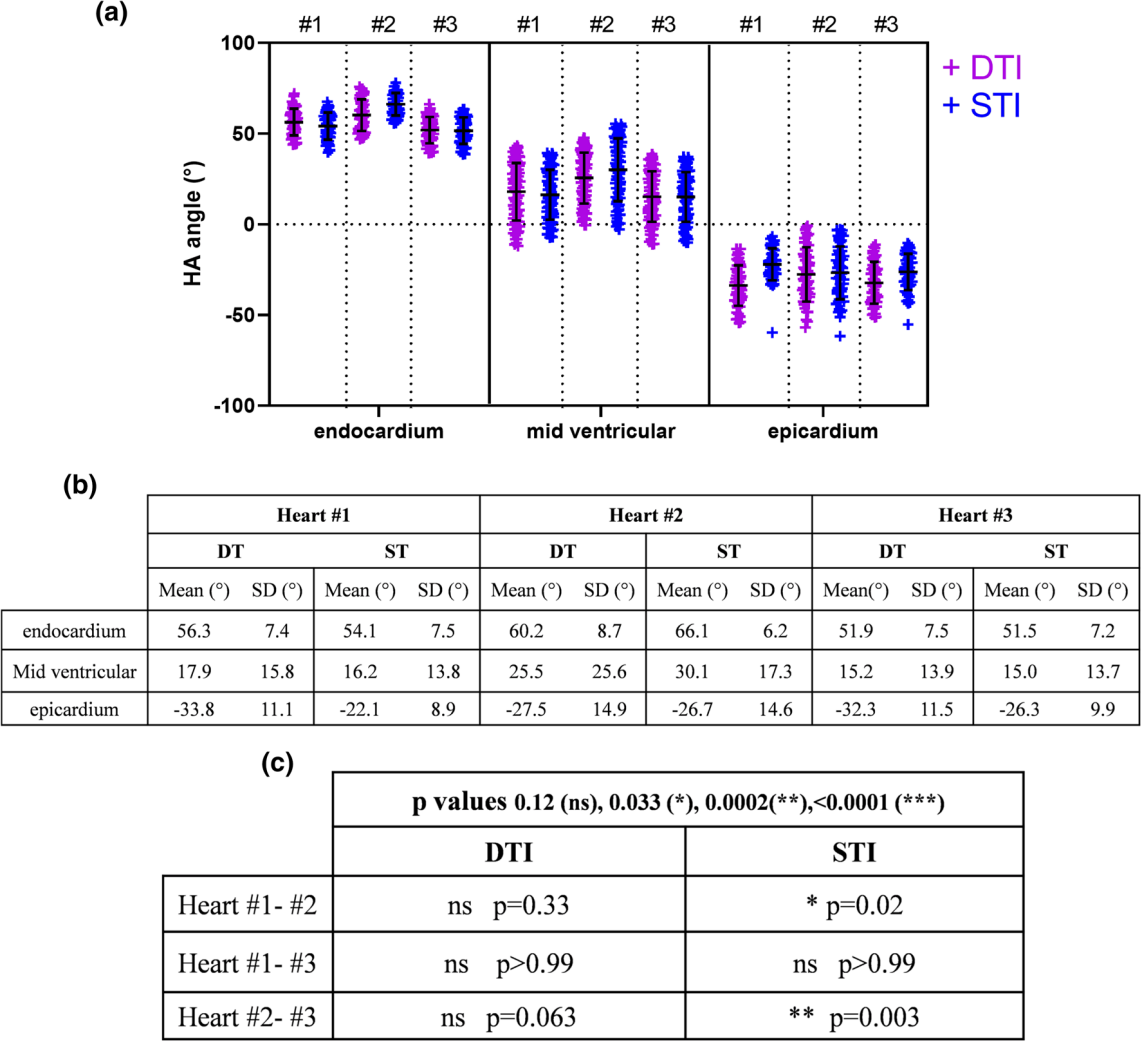
**a** Scatter plot comparison of total mean for HA angles in degree (°) divided in three areas along cardiac muscle (endocardium, mid-ventricular and epicardium) between DT and ST and between the three hearts. **b** Table of HA mean and SD for each heart along myocardium for hearts #1, #2 and #3. **c** Statistical results between three hearts and between STI and DTI methods

A more quantitative approach is described in the next Fig. 6. Global transmural profiles for mean and standard deviation of HA are presented in scatter plots in Fig. 6a for DTI and STI. The table in Fig. 6b presents mean and standard deviation for each heart in three areas: endocardium, mid-ventricular and epicardium for the two methods.

In the endocardium, the mean of HA is in the same range for DT with difference in mean of 3.9 ± 16.1° between hearts #1 and #2, 8.3 ± 16.2° between hearts #2 and #3 and 4.4 ± 14.9° between hearts #1 and #3. STI displays differences in mean of 12 ± 13.7°, 14.6 ± 13.4° and 2.6 ± 14.6° between hearts #1 and #2, between hearts #2 and #3 and between hearts #1 and #3, respectively.

In the mid-ventricular area, heart #2 displays higher values in DT (25.5 ± 25.6°) and ST (30.1 ± 17.3°) for HA angle in comparison with heart #1 and heart #3. In this area, higher standard deviation is calculated due to the transition of positive to negative HA angles. Then, in the epicardium, we can see that HA from DTI displays lower values (between − 27.5 ± 14.9° and − 33.8 ± 11.1°) than for STI (values between − 22.1 ± 8.9° and − 26.7 ± 14.6°). Statistical results are presented in in Fig. 6c between hearts for STI and STI. For the DTI method, no significant difference is observed with a global *p* > 0.12 between hearts #1 and #2, hearts #1 and #3 and hearts #2 and #3. For the STI method, the differences between samples are more pronounced: with a *p* = 0.02 and *p* = 0.003, respectively, for hearts #1 and #2 and hearts #2 and #3; no significant difference is observed in STI between hearts #1 and #3.

**Fig. 7:**
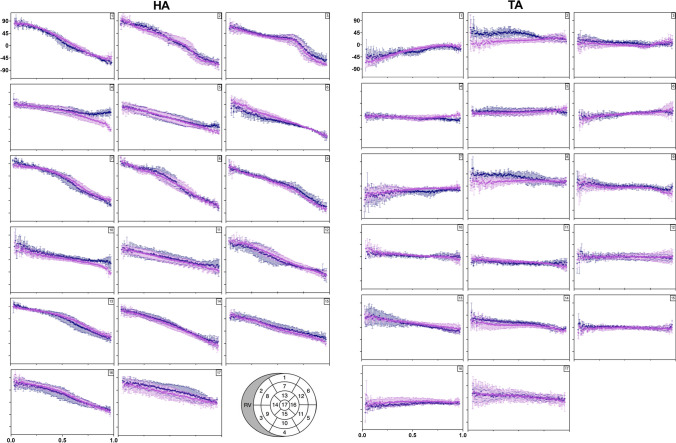
Transmural variation in myocytes orientations comparison between mean of DTI and STI for all hearts. Regions were defined by the 17-segment AHA model. Segments 1–17 are presented for DTI (violet dots) and STI (blue dots) with respective linear fit (lines). Helix angle (HA) and transverse angle (TA) are displayed. Mean of HA and TA for three hearts is shown between − 100° and 100° in *y* axis in function of LV wall thickness normalized between 0 and 1 corresponding to the LV epicardium and endocardium, respectively, in *x* axis

Transmural evolution for mean and standard deviation of HA and TA angles for three hearts are plotted between − 90° on the endocardium to 90° on the epicardium in Fig. 7 spread over 17 segments AHA model for DTI and STI (in violet and blue on the graphs, respectively). Their linearity and slope coefficient slope from LV endocardium to epicardium are summarized in Table 1. Experimental plots of angles between the endocardium and epicardium were remarkably consistent between STI and DTI for the two angles profiles in all 17 segments. For HA angles profiles, the *R*^2^ calculated ranges from 0.63 to 0.97 using DTI and from 0.66 to 0.95 for STI. Across the 17 different regions, slopes means for HA are − 129 ± 34° and − 121 ± 43° for DTI and STI, respectively. Linearity for TA is more variable across segments with *R*^2^ = 0.53 ± 0.38 for DTI and for STI *R*^2^ = 0.61 ± 0.29. A difference in slope is noticeable between STI and DTI in segment 2 and 8 in RV/LV junction. A mean difference between DT and STI slopes for TA is 10.4 ± 5.7°, and we notice a higher difference for segment 2 of 50.1° (slope(DTI) = 21.3°, slope(STI) = − 28.8°) and for segment 8 of 41.5° (slope(DTI) = 4.8°, slope(STI) = − 36.7°) between DTI and STI.

**Table 1:** *R*^2^ and slope values (indicating linearity) of DTI and STI measures representing fiber orientation (HA, TA, SE nd SA angles)

SEG	Helix angle				Transverse angle			
	Linearity		Slope (°)		Linearity		Slope (°)	
	DTI	STI	DTI	STI	DTI	STI	DTI	STI
1	0.97	0.95	− 162.2 ± 1.2	− 172.9 ± 1.6	0.89	0.82	70.5 ± 1.7	45.8 ± 1.5
2	0.81	0.95	− 181.8 ± 2.3	− 180.7 ± 1.6	0.60	0.64	21.3 ± 1.2	− 28.8 ± 1.5
3	0.82	0.83	− 139.4 ± 2.7	− 127.6 ± 2.4	0.004	0.65	1.4 ± 1.5	− 16.0 ± 0.8
4	0.81	0.64	− 86.77 ± 1.7	− 42.8 ± 1.3	0.02	0.82	1.76 ± 0.9	− 16.0 ± 0.5
5	0.87	0.80	− 103.0 ± 1.6	− 86.8 ± 1.7	0.68	0.10	15.0 ± 0.7	3.4 ± 0.8
6	0.94	0.89	− 131.8 ± 1.3	− 109.8 ± 1.5	0.82	0.78	33.3 ± 1.1	25.3 ± 0.9
7	0.92	0.94	− 153.2 ± 1.9	− 175.3 ± 1.7	0.81	0.52	29.4 ± 1.0	23.5 ± 1.6
8	0.94	0.93	− 179.9 ± 1.8	− 182.5 ± 2.0	0.10	0.81	4.8 ± 1.0	− 36.7 ± 1.3
9	0.95	0.91	− 161.0 ± 1.5	− 149.2 ± 1.8	0.64	0.73	− 25.7 ± 1.4	− 24.3 ± 1.0
10	0.83	0.70	− 78.3 ± 1.5	− 66.1 ± 1.8	0.85	0.53	− 31.1 ± 0.9	− 14.4 ± 0.9
11	0.80	0.66	− 84.8 ± 1.7	− 73.2 ± 2.2	0.90	0.45	− 27.2 ± 0.6	− 11.8 ± 0.9
12	0.92	0.85	− 146.6 ± 1.7	− 130.5 ± 2.2	0.004	0.03	0.2 ± 0.7	− 2.7 ± 1.1
13	0.93	0.92	− 121.0 ± 1.4	− 137.5 ± 1.7	0.96	0.96	− 47.5 ± 0.6	− 62.5 ± 0.8
14	0.94	0.95	− 141.8 ± 1.5	− 138.0 ± 1.4	0.77	0.88	− 28.5 ± 1.1	− 40.4 ± 1.0
15	0.95	0.66	− 108.1 ± 1.0	− 93.6 ± 1.6	0.02	0.30	1.9 ± 1.0	− 8.2 ± 0.9
16	0.92	0.85	− 128.0 ± 1.6	− 117.2 ± 2.0	0.14	0.55	6.4 ± 1.1	13.0 ± 0.9
17	0.63	0.66	− 80.7 ± 2.6	− 80.8 ± 2.4	0.79	0.75	− 27.4 ± 1.0	− 27.3 ± 0.7

*R*^2^ and slopes were measured from the endocardium to the epicardium (wall thickness normalized between 0 and 1) in 17 segments of the LV

**Fig. 8:**
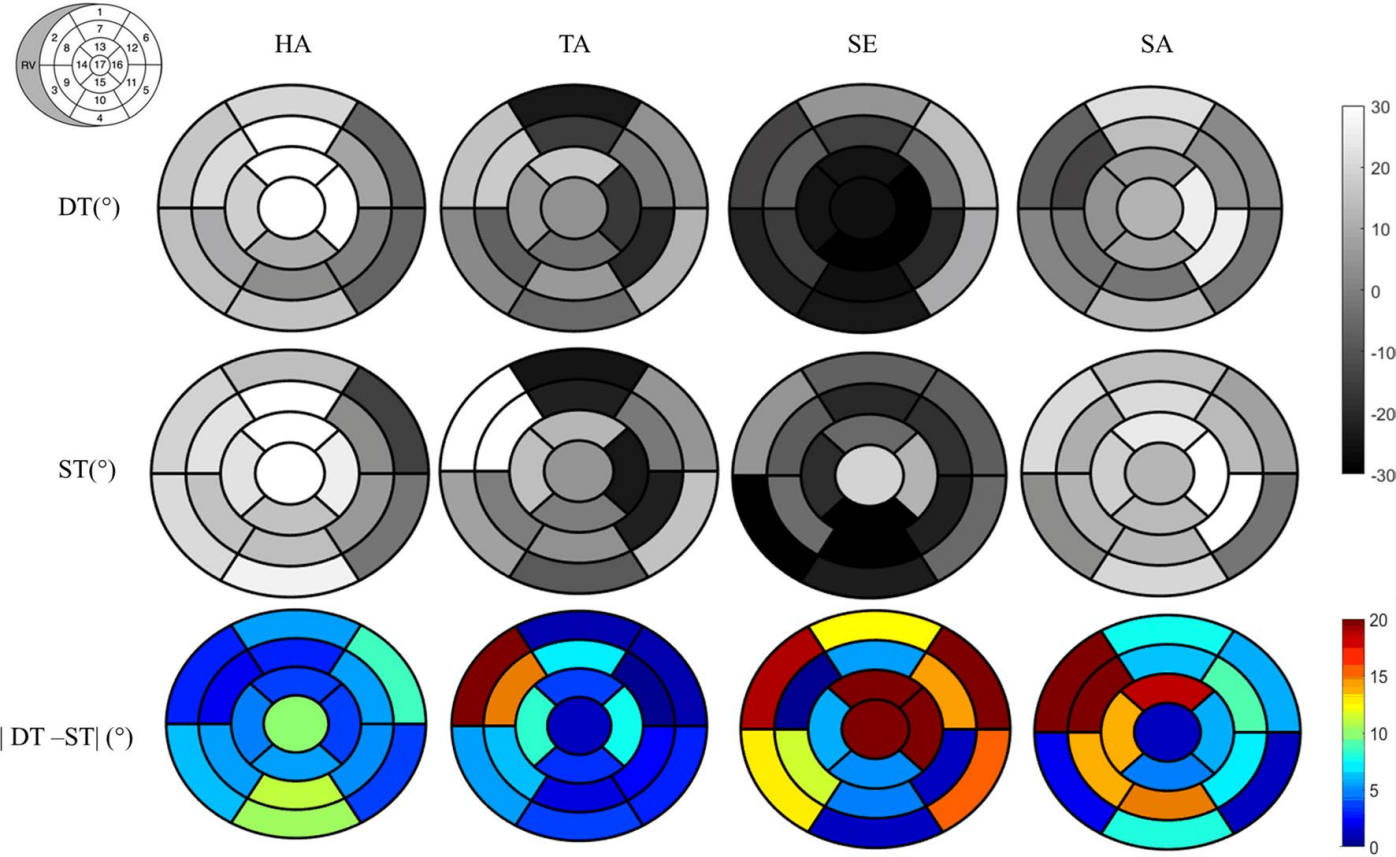
Seventeen segment distribution of mean of HA, TA, SE, SA angles for all hearts for DT and STI in degrees (°) along the left cardiac muscle is represented in gray between − 30° and 30°. The last line represents the absolute difference map between DTI and STI in color (range between 0° and 20°) for each angles in degrees (°)

Figure 8 displays mean values of HA, TA, SA and SE for 17 segments of the AHA model for DTI, STI and |DTI-STI| results for all hearts. HA distribution is consistent between DTI and STI; absolute differences between modalities are below 11° in all the 17 segments.

For TA distribution, difference maps between DTI and STI distribution are always below 10° except for segments 2 and 8 closed to RV with a difference higher than 15°. For SE, DTI displays globally negative values ranging for − 35.6° (segment 15) to 14.7° (segment 6). Absolute difference maps angles are close or above 20° in segments 6, 13, 16 and 17 (in red on SE difference maps with difference values of 22.5, 19.8, 42 and 45.4, respectively). For the SA angles, DTI and STI bullseye representation displays maps close to 0°. Absolute difference maps between DTI and STI displayed difference below 15° in absolute value except for in segments 2, 8, and 13 close to RV with difference of 27.9°, 24.4° and 18.4°, respectively, between |DTI-STI|.

**Fig. 9:**
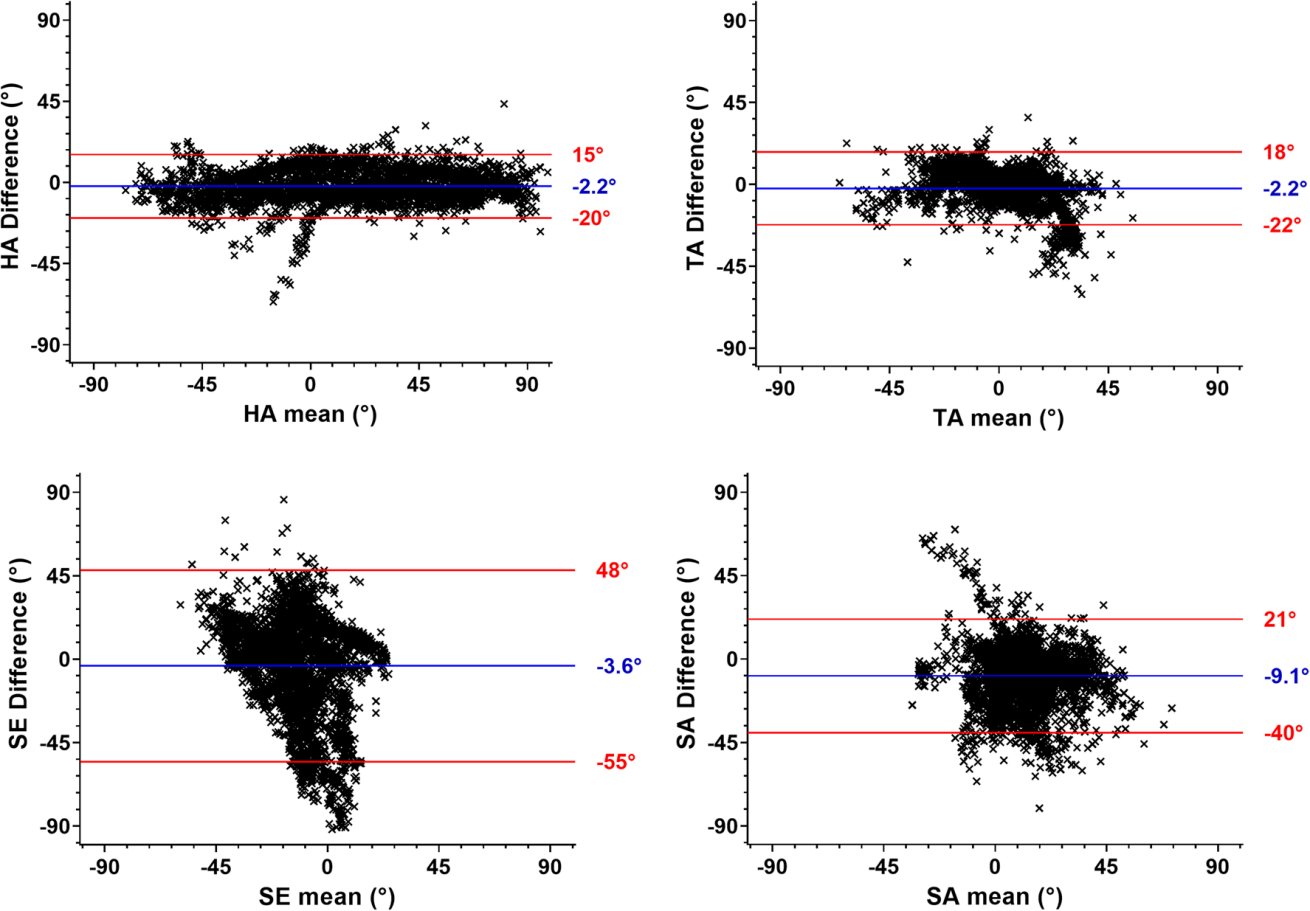
Bland–Altman plots of HA, TA, SE and SA angles measured with DTI and STI in the global left myocardium. Blue line represents the mean of angles difference between DT and ST (the bias) and red lines represent 95% limits of agreement as the mean difference (± 1.96 SD)

Figure 9 presents Bland–Altman plots for all three hearts for HA, TA, SE and SA angles between DTI and STI method in the LV wall. HA are distributed between − 90° and 90° in *x* axis with a HA_DTI-STI_ of − 2.2 ± 17.5° (mean ± 1.96 standard deviation in *y* axis). TA mean varies between − 45° and 45° in *x* axis with TA_DTI-STI_ centered to zero of − 2.2 ± 20.0°. For sheetlet comparison, SE points tend to be negative in mean with a SE_DTI-STI_ of − 3.6 ± 51.5°. SA mean varies between − 45° and 45° in *x* axis with SA_DTI-STI_ centered to zero of − 9.1 ± 30.5°.

## Discussion

We presented a detailed investigation and comparison between DTI and STI on several hearts from a large mammalian species (sheep) with same age, size, and cardiac fixation and without any cardiac pathologies, to study robustness of STI acquisition and post processing on whole hearts. This allowed us to successfully analyze myocardial structure using a combination of 3D STI (150–600 µm) and 3D DTI (600 µm) within a total acquisition time of ~ 2 days.

In the current study, we have used the same acquisition and a similar post processing pipeline as those presented in the 2019 FIHM conference [20]. However, in this previous publication, STI and DTI are only briefly compared for one human heart. Access to human hearts is limited and does not allow performing a thorough analysis of the performance and robustness of the proposed methods. The novelty of the current manuscript relies on the detailed study of the robustness of cardiac sample preparation, acquisition parameters and results obtained by DTI and STI that goes well beyond the scope of preliminary data presented in our previous conference proceedings [20].

The fibers followed the widely described organization with transmural fiber rotation; whereas, sheetlets in the three ventricles appeared macroscopically to be mainly aligned along two distinct perpendicular orientations across the LV wall. Previous studies have demonstrated the utility of DTI [11, 13–15, 29, 30] to quantify fiber orientation in the heart. Smooth fiber angles can be seen in DTI scans, and observations from the images have already been validated using histology [31]. DTI acquisition could be improved by adding more directions (> 6) which is already described in published works [11, 12]. In this study, we used NA = 1 and 6 directions, which is the minimum to calculate the tensor from diffusion-weighted imaging data. However, we obtained smooth data and reproducible results on three ex vivo sheep hearts with good image quality (see Fig. 3). Nevertheless, the measure is indirect (water diffusion using one compartmental model), and DTI has been shown to allow only limited assessment of laminar directions [32]. With HR MRI, Gilbert et al. demonstrated that there was very good consistency between contrast in native HR images and histology in terms of following the architecture of myolaminae [18]. Acquisition of HR images can be time consuming (more than 24 h) and post-processing is challenging. However, we have recently shown that HR MRI combined with STI analysis can yield robust information on the orientation of cardiac sheetlets. To date, this has only been performed in small animal hearts, mainly due to hardware limitations. To acquire in 3D an isotropic resolution of 150 µm could be limited by gradient strength and SNR on conventional MRI [18, 19].

### Comparison between DTI and STI

We demonstrated that the DTI and STI myofiber orientation exhibited similar rotation along the short and long axes, and both imaging modalities produced analogous maps of fiber orientation consistent with a previous study on rat hearts [19]. HA maps derived from both DTI and STI exhibited a smooth transmural orientation, with very high linear correlation coefficients. TA maps were close to 0° for both acquisitions. Cardiac fiber orientation was well correlated between STI and DTI.

FLASH images at 150 µm on sheep hearts at 9.4 T includes a lot of information like vessels, fiber and sheet orientation, etc. We noticed that on FLASH images, we can both identify bright and black interstices in specific area. The black interstices’ meaning remains speculative, but it could indicate either less well-perfused regions during the fixation protocol or larger extracellular clefts where less Gd is accumulated. However, it probably did not affect quantitative analysis, given the good correspondence between DTI and STI (at least in fiber orientation).

Bland–Altman (Fig. 9) plots assess the correspondence of DTI and STI measurements across the whole three hearts. These plots allow to compare the same measurements using two different techniques [33]. For fiber orientation, distribution for HA demonstrated no difference between − 60° and 60° across the left ventricle. For TA angles, we showed that the mean of TA is close to 0° across the myocardium, and a small bias is observed between two techniques. These results are in good agreement with the 17-segment representation, where the absolute difference in all segments is close to zero for HA and TA angles (see Fig. 8). Segments correlation in the AHA model has resulted in difference maps close to 0° for fibers structure. For the SE and SA angles extracted from DTI and STI, a large bias between techniques is noticed (see Fig. 8), indicating that SE and SA angles are larger for STI measurements that for DTI measurements (see also graphs for SE as SA in supplementary material S1). Indeed, for laminar structure, bullseye presentation displays a more heterogeneous differences for the two techniques. A frequent area with large differences between techniques appears in the LV/RV junction segments 2, 7 and 8. This region presents an underlying structure between the right and left ventricles. In Fig. 9, for SE, the 95% confidence interval is between 48° and − 55°, for SA between 21° and − 40°. These values are larger than for HA differences, between 15° and − 20°, and TA differences between 18° and − 22°.”

Overall, the differences between STI and DTI were larger for SE and SA (~ 6.3°) than for HA and TA (~ 2.2°). Similar results were found by Bernus et al. [19], showing in rat hearts differences up to 20° between DTI and STI sheetlet orientation due in part to eigenvectors sorting issues.

### Limitation: fixation process

Mazumder et al. [34] demonstrated that formalin fixation affects molecular diffusivity by reducing FA and mean fiber length and increasing ADC. It results in structural changes including shrinkage of sheetlet interstices, and consequently, sheetlet orientations may not reflect in vivo sheetlet measurements. However, fixation does not alter the structural orientation of the fibers. This hypothesis is further supported by another study where 4 weeks of formalin fixation with a 10% neutral buffered solution resulted in decreased diffusivity in mouse brains [35]. Moreover, the type of fixation (perfusion fixation, immersion fixation or both), thickness of tissue, preparation of the fixing agent, time interval between tissue extraction and fixation, temperature of fixation, play a role in the fixation process which can affect the diffusion properties of the heart differently. In comparison, protocols for in vivo diffusion MRI are improving, but they remain resolution-limited and prone to artifacts from respiratory, cardiac motion and strain [36, 37]. Moreover, fixation before imaging fixed hearts which do not represent truly systolic or diastolic phase; a direct comparison with in vivo measurements is complicated because state of ex vivo hearts is not matched to in vivo contractile state. Pennell’s group [9, 37] measured limited changes for fibers orientation (HA angles) between in vivo cardiac phase (systolic and diastolic) and ex vivo states (relaxed and contracted). Whereas for sheetlets arrangement, they demonstrated that results are dependent on contractile state.

### Limitation temperature

In this study, the three hearts were perfusion fixed in 10% formaldehyde (total fixation solution of 1 L) containing 2 mL of gadoterate meglumine (Gd Dotarem), during 12 h. Then, hearts were immersed inside container with perfusion solution and were stored in a cold room. Scanner room temperature was monitored by constant air temperature around 19 °C. For three hearts the same protocol of fixation and storage have been applied (see material and method). During acquisition, an optical temperature probe is placed close to the sample and connected to a computer, the fiber monitors change of temperature. We measure an increase of temperature of 2° ± 0.5 °C using SE DTI for 6 h of recording. No temperature variation was measured during FLASH acquisition.

### Limitation B0 and B1

The acquisition of data from large-sized samples using ultra-high field HR MRI is still challenging due to B0 and B1 homogeneities. B0 could influence MR signal and create susceptibility artifacts and affect image quality. In this study, we used fomblin to avoid susceptibility effects in ventricular cavities. A 3D B0 mapping was performed, using a dual-echo steady-state sequence and performed a global shim to reach a full-width at half-maximum of 50 Hz. B1 could influence RF pulses efficiency and create signal drop-off associated with RF coil sensitivity. To achieve homogenous B1 field and excitation, B1 maps were acquired within 20 min and a shim box in 3D was placed in the septum. Local B1+ shimming was performed by determining a set of transmit phases and amplitudes that will maximize the homogeneity within a region of interest and avoid RF destructive effects. Moreover, in post-processing pipeline, a N4 filter was also used to correct low frequency intensity non-uniformity present in 3D volumes (due to coil sensitivity or B1 effect). B0 and B1 have been optimized for each acquisition. However, some studies present results a study based on SNR and the impact of DTI results on large mammalian hearts at 3 T and 7 T [12] and on ex vivo rat heart [38]; comparing different SNR and their impact on DTI and STI acquisition is outside the scope of this work.

### Limitation post processing on HR MRI images

A large amount of data produced by HR 3D imaging require adequate processing hardware and dedicated software. The acquisition time to obtain 3D images of the whole hearts is several hours, and if data for both DTI and STI are acquired, the total acquisition time could exceed several days (acquisition time in this study was 2 days). Moreover, Teh et al. [33] used tensor analysis on synchrotron radiation imaging; they investigated vessel segmentation for STI data processing. They presented better sheetlet definition on SE and SA angles maps. A limitation of our approach is, therefore, the post-processing pipeline of STI, which is both complex and time consuming, but essential to reduce noise and avoid potential reconstruction errors. Indeed, statistical analysis have shown a less robust results between hearts than for DTI (see Fig. 6).

MRI allowed unprecedented visualization of cardiac structure of the ex vivo sheep heart with a high level of structural accuracy. In particular, new areas of interest, such as the RV and RV/LV junction, can be observed directly (segments 2 and 8). We also noticed that angles in the RV present abrupt orientation changes. Indeed, the importance of using an appropriate coordinate system to investigate the RV fiber and sheetlet orientations, as using an LV centroid may give rise to artifactual measurements in fiber helix/transverse angles and sheetlet orientations [30, 39].

In conclusion, results of STI on three whole ex vivo sheep hearts demonstrated a good correspondence with DTI and opens new perspectives for HR 3D structural characterization of normal/pathological cardiac structure. These results applied on healthy and pathological hearts will provide new insight for cardiac modeling research groups and it will improve developments of mathematical cardiac model. The objective is to gain a better understanding of the links between structural remodeling and electrical disorders of the heart. These results hold promise for the development of new noninvasive imaging methods to better characterize the cardiac microstructure in healthy and pathological human hearts.

## Supplementary Information

Below is the link to the electronic supplementary material.

Supplementary file1. S1: Transmural variation in sheetlet orientations comparison between mean of DTI and STI for all hearts. Regions were defined by the 17-segment AHA model. Segments 1–17 are resented for DTI (violet dots) and STI (blue dots). Helix angle (HA) and Transverse angle (SA) are displayed. Mean of SE and SA for three hearts is shown between − 100° and 100° in *y* axis in function of LV wall thickness normalized between 0 and 1 corresponding to the LV epicardium and endocardium, respectively, in *x* axis. (TIF 78667 KB)

## Abbreviations

3D: Three-dimensional
DTI: Diffusion tensor imaging
FA: Fractional anisotropy
FLASH: Fast low angle shot
FOV: Field of view
Gd Dotarem: Gadolinium-based contrast agent (Dotarem)
HA: Helix angle
HR MRI: High-resolution magnetic resonance imaging
LV: Left ventricle
MRI: Magnetic resonance imaging
ROI: Region of interest
RV: Right ventricle
SA: Sheet azimuth
SE: Sheet elevation
STI: Structure tensor imaging
TA: Transverse angle
